# Circuit- and laminar-specific regulation of medial prefrontal neurons by chronic stress

**DOI:** 10.1186/s13578-023-01050-2

**Published:** 2023-05-18

**Authors:** Wei-Zhu Liu, Chun-Yan Wang, Yu Wang, Mei-Ting Cai, Wei-Xiang Zhong, Tian Liu, Zhi-Hao Wang, Han-Qing Pan, Wen-Hua Zhang, Bing-Xing Pan

**Affiliations:** 1 Department of Biological Science, School of Life Science, Nanchang University, Nanchang, 330031 China; 2 Laboratory of Fear and Anxiety Disorders, Institutes of Life Science, Nanchang University, Nanchang, 330031 China; 3 Jiangxi Provincial Key Laboratory of Interdisciplinary Science, Nanchang University, Nanchang, 330031 People’s Republic of China

**Keywords:** Chronic stress, Synaptic transmission, Neuronal circuit, Prefrontal cortex, Amygdala, Anxiety

## Abstract

**Background:**

Chronic stress exposure increases the risk of mental health problems such as anxiety and depression. The medial prefrontal cortex (mPFC) is a hub for controlling stress responses through communicating with multiple limbic structures, including the basolateral amygdala (BLA) and nucleus accumbens (NAc). However, considering the complex topographical organization of the mPFC neurons in different subregions (dmPFC vs. vmPFC) and across multiple layers (Layer II/III vs. Layer V), the exact effects of chronic stress on these distinct mPFC output neurons remain largely unknown.

**Results:**

We first characterized the topographical organization of mPFC neurons projecting to BLA and NAc. Then, by using a typical mouse model of chronic restraint stress (CRS), we investigated the effects of chronic stress on the synaptic activity and intrinsic properties of the two mPFC neuronal populations. Our results showed that there was limited collateralization of the BLA- and NAc-projecting pyramidal neurons, regardless of the subregion or layer they were situated in. CRS significantly reduced the inhibitory synaptic transmission onto the BLA-projecting neurons in dmPFC layer V without any effect on the excitatory synaptic transmission, thus leading to a shift of the excitation-inhibition (E-I) balance toward excitation. However, CRS did not affect the E-I balance in NAc-projecting neurons in any subregions or layers of mPFC. Moreover, CRS also preferentially increased the intrinsic excitability of the BLA-projecting neurons in dmPFC layer V. By contrast, it even caused a decreasing tendency in the excitability of NAc-projecting neurons in vmPFC layer II/III.

**Conclusion:**

Our findings indicate that chronic stress exposure preferentially modulates the activity of the mPFC-BLA circuit in a subregion (dmPFC) and laminar (layer V) -dependent manner.

**Supplementary Information:**

The online version contains supplementary material available at 10.1186/s13578-023-01050-2.

## Background

Exposure to extreme or prolonged environmental stress is widely recognized as a major risk in the pathogenesis of neuropsychiatric disorders, including anxiety and major depressive disorder [1–3]. Previous studies have consistently shown that the medial prefrontal cortex (mPFC) acts as a critical node in regulating stress-related anxiety behavior through its top-down control over the subcortical structures [4–6]. For instance, stress enhances the excitatory synaptic transmission from the mPFC to the amygdala and facilitates the expression of anxiety-like behavior in mice [7, 8].

The topographical and functional organization of the mPFC are complicated. mPFC is classically divided into dorsal mPFC (dmPFC) and ventral mPFC (vmPFC) in rodents based on cytoarchitectural differences [9]. It has been reported that dmPFC and vmPFC have opposing roles in stress reactivity and alcohol drinking [10, 11]. There is also evidence showing that dmPFC and vmPFC exhibit distinct roles in tuning the anxiety-like behavior, with dmPFC activation producing anxiety-like behavior, whereas vmPFC activation has no effect [12, 13]. Moreover, the rodent mPFC displays laminar organization and can be divided into multiple layers (layer I, II/III, V). Previous studies have also highlighted layer-specific responses of mPFC neurons to stress [14–17]. For example, chronic stress decreased the dendritic branching of the projection neurons in layer II/III, but increased it in layer V [18]. Another study also revealed that chronic stress produced synapse loss only in deeper layers [19]. Moreover, evidence shows layer-specific changes undergoing chronic pain, with enhanced activity in layer II/III neurons and decreased activity in layer V neurons [20].

The mPFC neurons send their projections to multiple cortical and subcortical regions. Among them, the basolateral amygdala (BLA) and nucleus accumbens (NAc), two regions critically involved in the regulation of stress response, receive relatively dense inputs from mPFC [21–24]. The mPFC-BLA circuit has been shown to play a crucial role in the pathophysiology of stress-related diseases [7, 8, 25, 26]. For instance, in stressed mice, the excitatory synaptic transmission in mPFC-BLA circuit is markedly increased, and this change is tightly correlated with the increased anxiety-like behavior [8]. The projection from mPFC to NAc, on the other hand, is generally thought as a reward circuit, and optogenetic activation of this circuit increases resilience against stress [27, 28]. Notably, while most of the previous work studied these two mPFC circuits as a whole, little is known about how the subcircuits established by the mPFC neurons in different subregions or layers respond in face of chronic stress.

To address this issue, we first characterized the topographical organization of mPFC neurons projecting to BLA and NAc. We then investigated the influence of chronic restraint stress on the synaptic and neuronal activity of the BLA- versus NAc-projecting neurons in different layers of dmPFC and vmPFC. Our results showed that CRS preferentially shifted the E-I balance toward excitation in BLA-projecting neurons in dmPFC layer V without any noticeable effect on NAc-projecting neurons, regardless of the subregions or layers. Moreover, CRS markedly increased the intrinsic excitability in BLA-projecting dmPFC neurons in layer V, but instead caused a tendency of decrease in NAc-projecting neurons in vmPFC layer II/III. Our findings thus suggest that the mPFC-BLA circuit is more vulnerable to chronic stress than its mPFC-NAc counterpart.

## Results

### Anatomical characterization of the BLA and NAc-projecting mPFC neurons

**Fig. 1:**
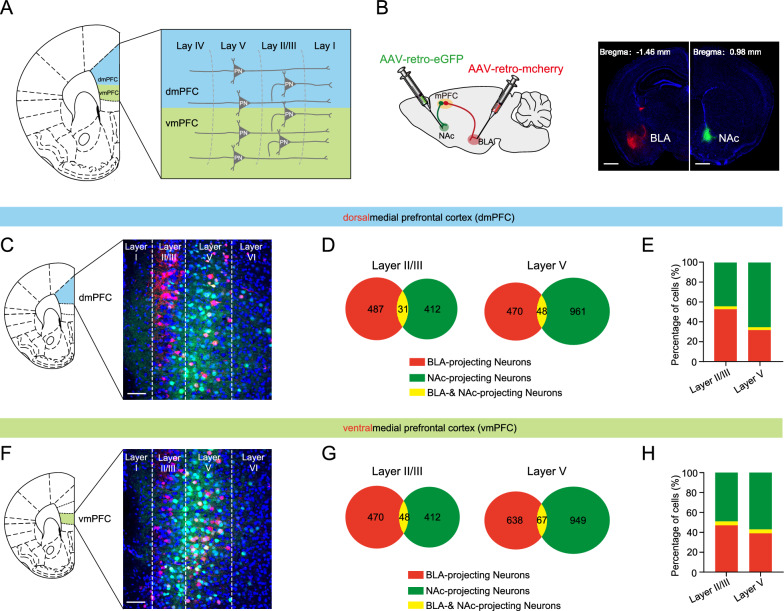
Anatomical characterization of the mPFC neurons projecting to the BLA and NAc. **A** Schematic structure of different regions and layers of the medial prefrontal cortex (mPFC). **B** Schematic showing injection of AAV2/retro-hSyn-eGFP into NAc and AAV2/retro-hSyn-mCherry into BLA (left) and representative images showing the injection site in BLA and NAc (right). Scale bar: 500 μm. **C** Representative images showing the labeled dmPFC^→BLA^ and dmPFC^→NAc^ PNs. Scale bar: 100 μm. **D** Pie charts illustrating the abundance of the BLA-and NAc-projecting neurons in dmPFC. **E** Bar graph illustrating the percentage of the BLA- and NAc-projecting neurons in dmPFC. **F** Representative images showing the labeled vmPFC^→BLA^ and vmPFC^→NAc^ PNs. Scale bar: 100 μm. **G** Pie charts illustrating the abundance of the BLA- and NAc-projecting neurons in vmPFC. **H** Bar graph illustrating the percentage of the BLA- and NAc-projecting neurons in vmPFC

To characterize the topographical organization and collateralization of the mPFC neurons projecting to BLA or NAc (Fig. 1A), we used a retrograde adeno-associated viral (AAV) tracing strategy by injecting retrogradely traveling AAV vector carrying mCherry (AAV-mCherry) and EGFP (AAV-EGFP) under the control of hSyn promoter into BLA and NAc, respectively (Fig. 1B). We found a similar proportion of neurons projecting to BLA (dmPFC^→BLA^ PNs) and NAc (dmPFC^→NAc^ PNs) in layer II/III, whereas in layer V, a higher proportion of dmPFC^→NAc^ PNs than dmPFC^→BLA^ PNs. Among the fluorescently labelled neurons, there were only few projecting to both regions in layer II/III and V (Fig. 1C–E). Similarly, the two neuronal populations were also found to be intermingled but seldom overlapped in layers II/III and V of vmPFC (Fig. 1F–H). These results indicate minimal collateralization of BLA- and NAc- projecting neurons in mPFC. We then determined the identity of the two long-range projection neuronal populations. Quantitative analysis revealed the presence of CaMKIIα (a pyramidal neuronal marker) in 96% of mCherry^+^ and 98% of EGFP^+^ cells, indicating most of the BLA- and NAc- projecting neurons in mPFC are glutamatergic cells (Additional file 1: Fig. S1).

### CRS selectively disrupts the excitation-inhibition balance of dmPFC^→BLA^ PNs in layer V

**Fig. 2:**
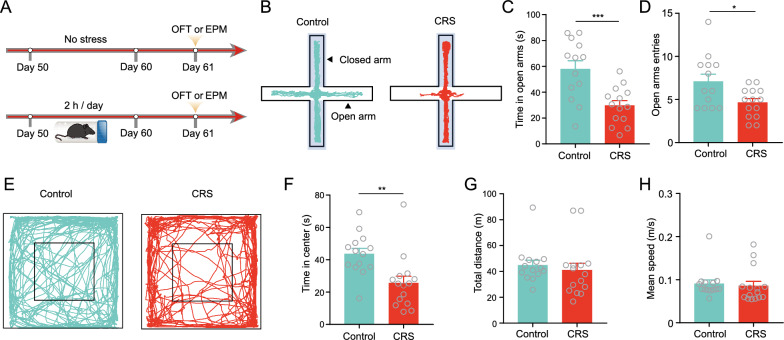
CRS significantly induces anxiety-like behavior in mice. **A** Experimental procedures. **B** Representative activity tracking in EPMT. **C**, **D** Summary plots of time in open arms (**C**) and open-arm entries (**D**) during EPMT. **E** Representative activity tracking in center area OFT. **F** Summary plots of time in center area during OFT. **G** Summary plots of total distance travelled during OFT. **H** Summary plots of mean speed during OFT

We next asked how the two neuronal populations responded to stress. We utilized chronic restraint stress (CRS) paradigm to induce anxiety-like behavior. Consistent with previous findings [8, 29, 30], the mice subjected to CRS displayed a typical anxiogenic phenotype as evidenced by less time in and entries into open arms during elevated plus maze test (EPMT) (Fig. 2A–D), as well as shorter time in the center area during open field test (OFT) than the non-stressed ones (Fig. 2E, F). However, there were no significant between-group changes in the total distance traveled and averaged speed, indicating that CRS does not alter locomotor activity (Fig. 2G, H).

**Fig. 3:**
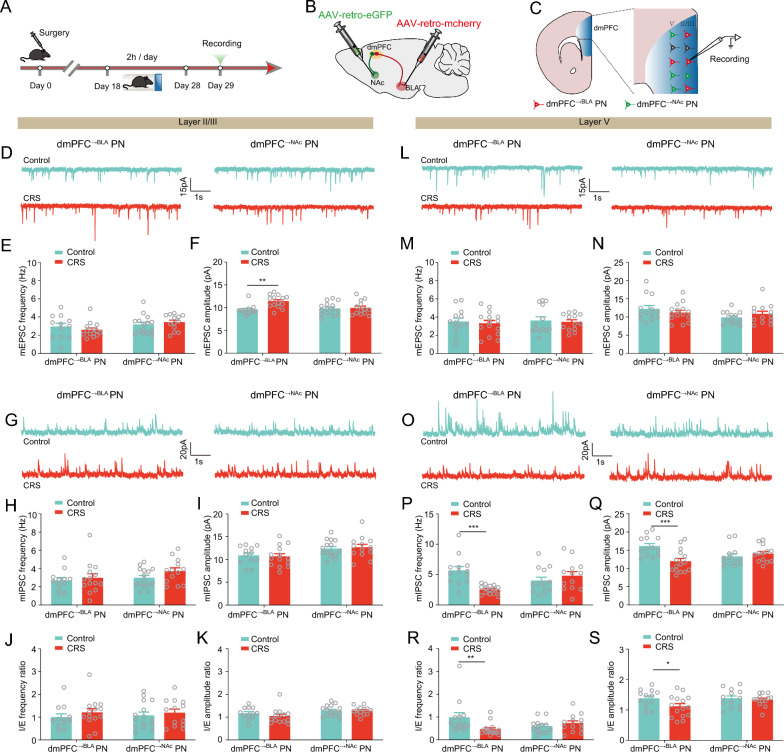
CRS markedly decreases inhibitory synaptic transmission onto dmPFC^→BLA^ PNs in layer V. **A** Experimental procedures. **B** Schematic showing injection of AAV2/retro-hSyn-eGFP into NAc and AAV2/retro-hSyn-mCherry into BLA. **C** Schematic showing recording in dmPFC^→BLA^ or dmPFC^→NAc^ PNs. **D** Representative traces showing miniature excitatory postsynaptic currents (mEPSCs) in dmPFC layer II/III (scale bar: 1 s, 15 pA). **E**, **F** Summary plots of averaged mEPSC frequency (**E**) and amplitude (**F**). **G** Representative traces showing mIPSCs in dmPFC layer II/III (scale bar: 1 s, 20 pA). **H,** **I** Summary plots of averaged mIPSC frequency (**H**) and amplitude (**I**). **J** Summary plots of I/E frequency ratio in dmPFC layer II/III. **K** Summary plots of I/E amplitude ratio in dmPFC layer II/III. **L** Representative traces showing mEPSCs in dmPFC layer V (scale bar: 1 s, 15 pA). **M**, **N** Summary plots of averaged mEPSC frequency (**M**) and amplitude (N). **O** Representative traces showing mIPSCs in dmPFC layer V (scale bar: 1 s, 20 pA). **P**, **Q** Summary plots of averaged mIPSC frequency (**P**) and amplitude (**Q**). **R** Summary plots of I/E frequency ratio in dmPFC layer V. **S** Summary plots of I/E amplitude ratio dmPFC layer V

We then investigated the impact of CRS on the electrophysiological properties of these two mPFC neuronal populations using whole-cell patch-clamp recordings in acutely isolated ex vivo brain slices (Fig. 3A–C). Since the imbalance between excitatory and inhibitory synaptic neurotransmission is thought as a main factor causing stress-related anxiety [8], we first measured the effects of CRS on the miniature excitatory postsynaptic currents (mEPSCs) and miniature inhibitory postsynaptic currents (mIPSCs) in dmPFC layer II/III and V neurons. As shown in Fig. 3D–F, in layer II/III, the frequency of mEPSCs remained unaltered in both dmPFC^→BLA^ and dmPFC^→NAc^ PNs, while the amplitude was increased only in the former population. Notably, neither the frequency nor the amplitude of mIPSCs in the two populations was altered by CRS (Fig. 3G–I). Similarly, the ratios of IPSCs/EPSCs frequency and amplitude were comparable between the two groups after CRS (Fig. 3J, K). In layer V, mEPSCs changes were observed in neither population (Fig. 3L–N). By contrast, CRS significantly decreased the frequency and amplitude of mIPSCs in dmPFC^→BLA^ but not dmPFC^→NAc^ PNs (Fig. 3O–Q). As a consequence, the frequency and amplitude of the I/EPSCs ratio were markedly decreased in dmPFC^→BLA^ PNs (Fig. 3R, S), suggesting that CRS selectively shifts the excitatory/inhibitory (E/I) balance toward excitation in the former but not the latter population.

To further test whether the shift of E/I balance in the dmPFC^→BLA^ PNs in layer V also occurred in other stress paradigms, we repeated the experiments in mice experiencing chronic unpredictable stress (CUS). As expected, CUS markedly increased the anxiety-like behavior as measured by EPMT and OFT (Additional file 1: Fig. S2). Similarly, CUS decreased the frequency and amplitude of mIPSCs in dmPFC^→BLA^ PNs without any effects in dmPFC^→NAc^ PNs in layer V. By contrast, mEPSCs changes were observed in neither PN population irrespective of the layers (Additional file 1: Fig. S3A–L). Not surprisingly, in the dmPFC^→BLA^ PNs in layer V, CUS caused a marked reduction of the I/EPSCs frequency ratio and a decreasing tendency in their amplitude ratio (Additional file 1: Fig. S3M–P).

Altogether, these results consistently suggest that chronic stress preferentially disrupts the E/I balance in the dmPFC^→BLA^ PNs in layer V, supporting the notion that chronic stress dysregulates the synaptic transmission in the dmPFC neurons in a layer- and projection-specific manner.

### CRS does not affect the excitation-inhibition balance of vmPFC^→BLA^ and vmPFC^→NAc^ PNs

**Fig. 4:**
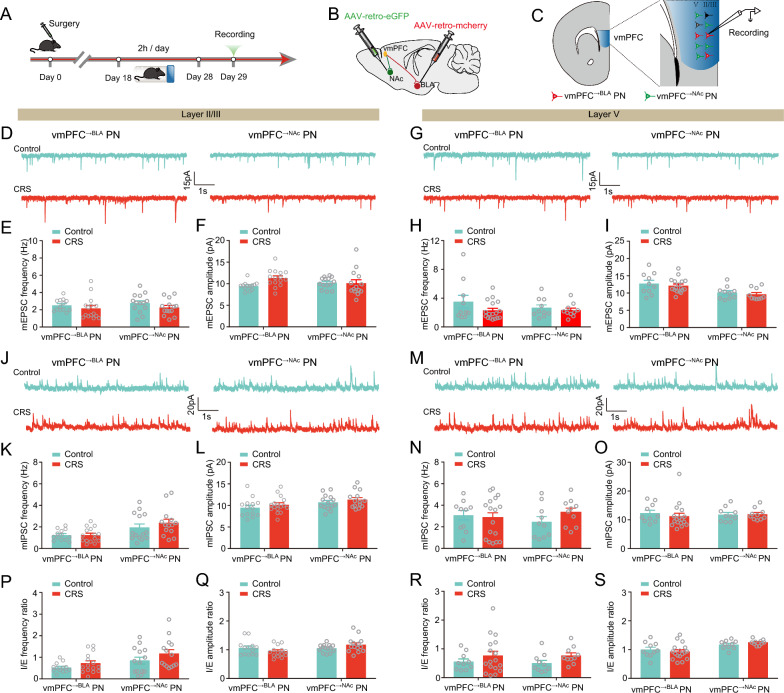
CRS does not affect the synaptic transmission onto both vmPFC^→BLA^ or vmPFC^→NAc^ PNs. **A** Experimental procedures. **B** Schematic showing injection of AAV2/retro-hSyn-eGFP into NAc and AAV2/retro-hSyn-mCherry into BLA. **C** Schematic showing recording in vmPFC^→BLA^ or vmPFC^→NAc^ PNs. **D** Representative traces showing mEPSCs in vmPFC layer II/III (scale bar: 1 s, 15 pA). **E**, **F** Summary plots of averaged mEPSC frequency (**E**) and amplitude (**F**). **G** Representative traces showing mEPSCs in vmPFC layer V (scale bar: 1 s, 15 pA). **H,** **I** Summary plots of averaged mEPSC frequency (**H**) and amplitude (**I**). **J** Representative traces showing mIPSCs in vmPFC layer II/III (scale bar: 1 s, 20 pA). **K**, **L** Summary plots of averaged mIPSC frequency (**K**) and amplitude (**L**). **M** Representative traces showing mIPSCs in vmPFC layer V (scale bar: 1 s, 20 pA). **N**, **O** Summary plots of averaged mIPSC frequency (**N**) and amplitude (**O**). **P** Summary plots of I/E frequency ratio in vmPFC layer II/III. **Q** Summary plots of I/E amplitude ratio in vmPFC layer II/III. **R** Summary plots of I/E frequency ratio in vmPFC layer V. **S** Summary plots of I/E amplitude ratio vmPFC layer V

Next, we explored how CRS would affect the E/I balance in vmPFC-BLA and vmPFC-NAc circuits (Fig. 4A–C). We found that neither the frequency nor the amplitude of mEPSCs was altered in the two PN populations in layer II/III and V (Fig. 4D–I). Similarly, the mIPSCs changes were observed in neither PN population (Fig. 4J–O), yielding unaltered I/EPSCs frequency or amplitude ratio in both populations (Fig. 4P–S). We repeated the above tests in CUS models and also found no mEPSCs and mIPSCs changes in both populations (Additional file 1: Fig. S4). Altogether, unlike its selective regulation of the E/I balance in BLA-projecting PNs in the layer V of dmPFC, chronic stress has a negligible effect on the vmPFC neurons disregarding whether they project to BLA or NAc.

### CRS selectively increases intrinsic excitability of dmPFC^→BLA^ PNs in layer V

**Fig. 5:**
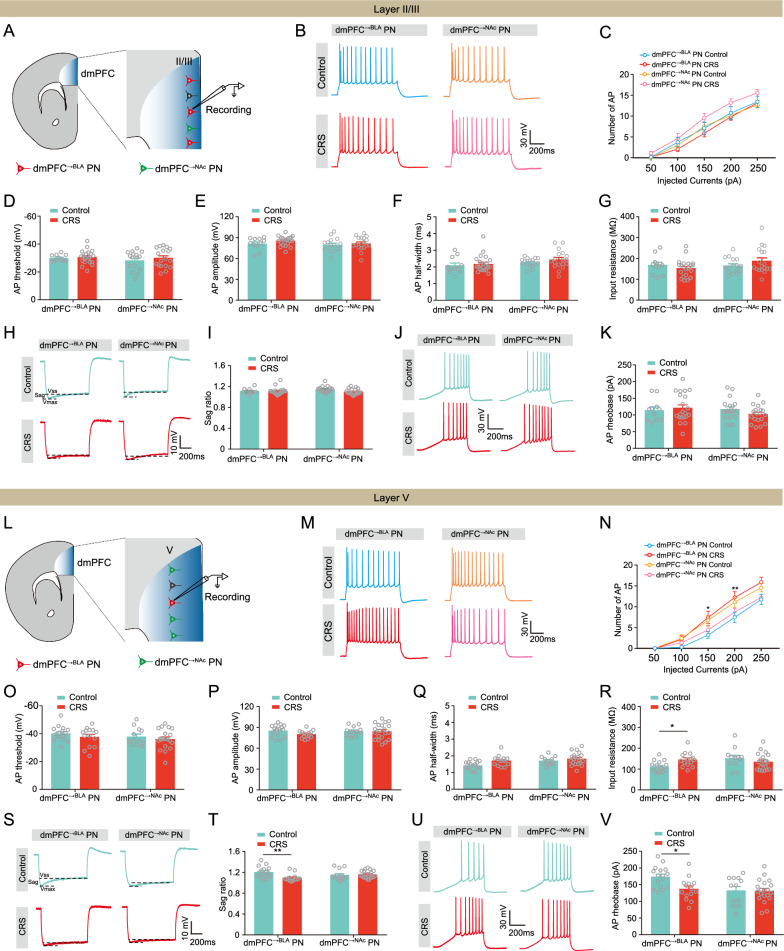
CRS markedly increases intrinsic excitability of dmPFC^→BLA^ PNs in layer V. **A** Schematic showing recording in dmPFC^→BLA^ or dmPFC^→NAc^ PNs in layer II/III. **B** Representative traces showing APs in dmPFC layer II/III (scale bar: 200 ms, 30 mV). **C** Summary plots of APs. **D** Summary plots of AP threshold. **E** Summary plots of AP amplitude. **F** Summary plots of AP half-width. **G** Summary plots of input resistance. **H** Representative traces showing sag in dmPFC layer II/III (scale bar: 200 ms, 10 mV). **I** Summary plots of sag ratio. **J** Representative traces showing rheobase in dmPFC layer II/III (scale bar: 200 ms, 30 mV). **K** Summary plots of rheobase. **L** Schematic showing recording in dmPFC^→BLA^ or dmPFC^→NAc^ PNs in layer V. **M** Representative traces showing APs in dmPFC layer V (scale bar: 200 ms, 30 mV). **N** Summary plots of APs. **O** Summary plots of AP threshold. **P** Summary plots of AP amplitude. **Q** Summary plots of AP half-width. **R** Summary plots of input resistance. **S** Representative traces showing sag in dmPFC layer V (scale bar: 200 ms, 10 mV). **T** Summary plots of sag ratio. **U** Representative traces showing rheobase in dmPFC layer V (scale bar: 200 ms, 30 mV). **V** Summary plots of rheobase

In addition to causing changes in synaptic activity, the external stimuli also regulate the neuronal activity through altering their intrinsic excitability [22]. We then examined the effect of CRS on the intrinsic excitability of the two PN populations in dmPFC by evoking the action potentials (AP) in these neurons (Fig. 5A). In layer II/III, CRS altered neither the number of AP in dmPFC^→BLA^ and dmPFC^→NAc^ PNs nor the parameters depicting AP including the threshold, amplitude, and half-width. Moreover, CRS failed to affect the input resistance, sag ratio, and rheobase in these neurons (Fig. 5B–K). However, in layer V, CRS selectively increased the number of APs in dmPFC^→BLA^ PNs but not dmPFC^→NAc^ PNs (Fig. 5L–N). We also analyzed the AP parameters, which have been widely proved to contribute to altering intrinsic excitability. CRS had no obvious effect on the threshold, amplitude, and half-width of AP of two PN populations (Fig. 5O–Q) but selectively increased the input resistance of the dmPFC^→BLA^ PNs (Fig. 5R). In contrast, the sag ratio and rheobase in the dmPFC^→BLA^ PNs were decreased following chronic stress (Fig. 5S–V).

Taken together, these results demonstrate that CRS preferentially increases the intrinsic excitability of the dmPFC^→BLA^ neurons but not their dmPFC^→NAc^ neighbors, and this effect appears to only emerge in layer V but not layer II/III. Thus, in line with its layer and projection-specific influence on the synaptic activity of dmPFC neurons, the effect of chronic stress on the intrinsic excitability of dmPFC neurons also varies with their projection targets and the layer they are located in. Relative to the dmPFC-NAc circuit, the dmPFC-BLA circuit appears more vulnerable to stress exposure.

### CRS does not alter intrinsic excitability of vmPFC^→BLA^ and vmPFC^→NAc^ PNs

**Fig. 6:**
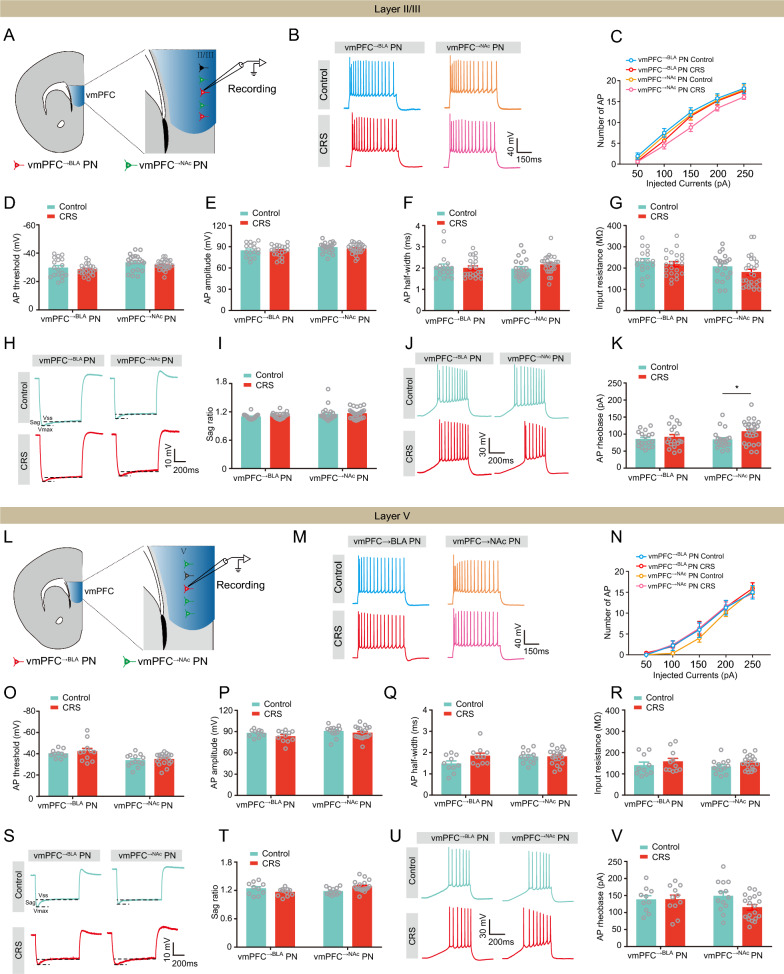
CRS does not alter the intrinsic excitability of both vmPFC^→BLA^ or vmPFC^→NAc^ PNs. **A** Schematic showing recording in vmPFC^→BLA^ or vmPFC^→NAc^ PNs in layer II/III. **B** Representative traces showing APs in vmPFC layer II/III (scale bar: 150 ms, 40 mV). **C** Summary plots of APs. **D** Summary plots of AP threshold. **E** Summary plots of AP amplitude. **F** Summary plots of AP half-width. **G** Summary plots of input resistance. **H** Representative traces showing sag in vmPFC layer II/III (scale bar: 200 ms, 10 mV). **I** Summary plots of sag ratio. **J** Representative traces showing rheobase in vmPFC layer II/III (scale bar: 200 ms, 30 mV). **K** Summary plots of rheobase. **L** Schematic showing recording in vmPFC^→BLA^ or vmPFC^→NAc^ PNs in layer V. **M** Representative traces showing APs in vmPFC layer V (scale bar: 150 ms, 40 mV). **N** Summary plots of APs. **O** Summary plots of AP threshold. **P** Summary plots of AP amplitude. **Q** Summary plots of AP half-width. **R** Summary plots of input resistance. **S** Representative traces showing sag in vmPFC layer V (scale bar: 200 ms, 10 mV). **T** Summary plots of sag ratio. **U** Representative traces showing rheobase in vmPFC layer V (scale bar: 200 ms, 30 mV). **V** Summary plots of rheobase

Having found that CRS had little influence on the synaptic activity of the two PN populations in vmPFC (Fig. 4), we next tested whether CRS would change their intrinsic excitability. The results showed that in layer II/III, the number of AP in vmPFC^→BLA^ PNs was not altered by CRS; however, there was a decreasing tendency in the vmPFC^→NAc^ PNs (Fig. 6A–C). The AP parameters, including the threshold, amplitude and half-width, and the input resistance of the two PN populations were unaffected by CRS (Fig. 6D–G). Additionally, CRS unaltered the sag ratio in both populations but preferentially increased the rheobase of vmPFC^→NAc^ PNs (Fig. 6H–K). In contrast, in layer V, neither the AP number nor the AP parameters were changed by CRS (Fig. 6L–V).

## Discussion

In the present study, we provide new evidence that chronic stress exposure dysregulates the synaptic activity and intrinsic excitability in distinct mPFC PN populations in a laminar- and subregion-dependent manner. For the mPFC^→BLA^ PNs, CRS caused a shift of the E-I balance of synaptic transmission toward excitation and an increase of neuronal intrinsic excitability, but only in those situated in layer V of dmPFC. Neither the vmPFC^→BLA^ PNs nor the d/vmPFC^→NAc^ PNs were affected following CRS exposure, suggesting that among the complex neuronal populations in mPFC, the dmPFC^→BLA^ PNs, particularly for those in layer V of dmPFC, represent a group vulnerable to chronic stress exposure.

As a critical hub for brain to cope with stress, mPFC is highly heterogenous in terms of the responses made by the neurons in different subregions or even different layers [4, 31, 32]. Mounting evidence indicates that the dmPFC and vmPFC neurons have distinct roles in stress coping and regulating anxiety and fear expression [12]. For instance, while the dmPFC neurons were shown to mainly participate in anxiety and fear expression, those in vmPFC are more engaged in fear extinction and have little effect on anxiety-like behavior [12, 33]. At a more microscopic level, chronic stress was observed to alter the spine density and increase the expression of c-fos, an indicator of neuronal activity, in dmPFC but not vmPFC neurons [11, 34]. Adding complexity to these, we found that CRS also preferentially decreased the inhibitory transmission and increased intrinsic excitability in dmPFC but not vmPFC neurons. Although the exact reasons for the subregion-specific changes are not yet known, there are finding showing that the GABAergic transmission and neuronal intrinsic excitability differ between dmPFC and vmPFC. For the dmPFC neurons, they receive relatively stronger GABAergic inputs but have weaker intrinsic excitability than their vmPFC neighbors[35, 36]. These differences may be related to the different influences of CRS on these two mPFC PN populations. In addition to this, we further found that these CRS influences only occurred in the dmPFC layer V but not layer II/III neurons. It may not be surprising, given that increasing evidence has been accumulated to show that the superficial and deeper layers of mPFC neurons are differently affected by acute versus chronic stress. For instance, chronic stress was shown to alter the excitability and synaptic transmission in the mPFC layer V but not layer II/III neurons [16, 37]. By contrast, acute stress selectively altered the synaptic transmission onto mPFC neurons in layer II/III but not layer V neurons [17].

Recent studies have begun to show that individual mPFC neurons exhibit markedly different responses to stress, which is likely associated with their molecular profiles and structural connectivity with other brain regions. For instance, chronic unpredictable stress increases excitability and excitatory synaptic transmission of dopamine D1-type receptor-expressing pyramidal neurons in the dmPFC, but reduces that in dopamine D2-type receptor-expressing neurons [37]. In terms of structural connectivity, evidence shows that chronic variable stress (CVS) increases the activity of ventral tegmental area (VTA)-projecting mPFC neurons and induces anxiety-like behavior [38]. Optogenetic inhibition of the dorsal raphe nucleus projecting mPFC neurons reduces social behaviors after chronic social defeat stress (CSDS) [39]. In supporting the view that stress causes specific regulation of activity of mPFC neurons in a circuit-dependent manner, we here found that CRS selectively decreased the inhibitory synaptic transmission and increased the intrinsic excitability in dmPFC^→BLA^ neurons in layer V, which added new evidence to previous literature highlighting the importance of mPFC-BLA circuit in the pathology of stress-related disorders [7, 8, 40–42]. Notably, for the NAc-projecting mPFC neurons, we did not observe any changes in the synaptic transmission, except for a tendency of decrease in intrinsic excitability. We speculate that dmPFC^→BLA^ neurons may be more vulnerable to chronic stress than its dmPFC-NAc counterpart. It should be noted that we only used male mice in this study. A previous study showed that CVS causes sex-specific modifications in the morphology of NAc- projecting mPFC neurons, since CVS decreased the dendritic arborization in NAc-projecting neurons in female mice but not male mice [38]. Considering females are more sensitive to stress exposure and are more likely to develop anxiety and depression, it would be interesting to determine whether CRS has any effect on the NAc-projecting mPFC neurons in female in future studies.

Chronic stress-induced disruption of excitatory-inhibitory (E-I) balance in mPFC plays a critical role in the pathological of anxiety- and depression-like behavior [43, 44]. For example, a study showed that chronic stress reduces the inhibitory transmission in the mPFC [45]; interestingly, we found that the reduced inhibitory transmission mainly occurred in the dmPFC-BLA neurons but not in their proximal dmPFC-NAc ones. Since the excitatory synaptic transmission remained unaffected, a net shift of the E-I balance of synaptic transmission toward excitation emerged in dmPFC-BLA neurons. Although the exact molecular mechanisms underlie the CRS-induced decrease in the inhibitory signal in dmPFC-BLA neurons remains unknown, one possible explanation is that CRS may inhibit the probability of GABA release from presynaptic terminals of GABAergic interneurons. It has been showed that chronic stress decreases the release probability of GABA from GABAergic interneurons, leading to a decrease in the frequency of mIPSCs in mPFC [45]. On the other hand, as generally known, the GABAergic interneurons are composed of multiple populations which express relatively specific markers such as the calcium-binding protein parvalbumin (PV), the neuropeptide somatostatin (SST) and the vasoactive intestinal peptide (VIP) respectively with each providing distinct inhibition onto the pyramidal neurons [46–48]. Accumulating evidence suggests a causal relationship between the deficit of interneurons in mPFC and stress-related psychiatric disorders. For example, chronic stress reduces the cell density of PV interneurons in mPFC [49–51], and causes deficit of SST cell function [52]. It is worthwhile to identify which interneuron subtype contributes to the chronic stress-induced deficit of GABAergic signal in the BLA-projecting dmPFC neurons.

Except for the reduced inhibitory synaptic transmission, another important observation in the present study was that CRS also caused strengthened intrinsic excitability of BLA-projecting dmPFC neurons in layer V. This may provide an explanation for why CRS enhances the glutamatergic transmission from dmPFC projection to BLA neurons in our recent study [8]. One limitation of the study is that the potential factors contributing to the regulation of intrinsic excitability are not investigated. However, our further analysis of the AP parameters revealed that CRS increased the input resistance while decreased the rheobase and sag ratio in BLA-projecting dmPFC neurons, hinting a potential involvement of HCN channels, which have been widely reported in regulating the input resistance and sag ratio, and subsequently modulates neuronal excitability [36, 53–55], and are considered as a potential target for treating stress-related disorders, such as anxiety and depression [56, 57]. For instance, knockdown of HCN channels in the dorsal hippocampal increased neuronal excitability that is sufficient to produce anxiolytic-like and antidepressant effects [57]. In addition, chronic stress increases neuron firing through HCN channel impairments in BLA and induces anxiety-like behavior [58]. In line with our results that CRS enhanced the excitability of BLA-projecting neurons in dmPFC layer V but not in layer II/III, a previous finding shows that the HCN channel expression is higher in Layer V neurons than layer II/III [36], it is plausible that the different expression and function of HCN channels may provide a physiological basis for layer-specific response to chronic stress.

While our study showed for the first time that chronic stress differentially regulates the GABAergic transmission and intrinsic excitability of mPFC neurons projecting to BLA and NAc in a laminar- and subregion-dependent manner, some questions are still open. For example, although we have observed that CRS decreased inhibitory synaptic transmission and increased intrinsic excitability in BLA- but not NAc-projecting dmPFC neurons, what are the molecular mechanisms driving such changes? Second, the causal link between the CRS-induced dysregulation of functional neuroplasticity in dmPFC neurons projecting to BLA and anxiety-like behavior remains unknown. Answering these important questions will expand our understanding of the pathological mechanism of stress-related disorders.

## Materials and methods

### Animals

C57BL/6J mice were bred in the animal facility of Nanchang University. 4–5 mice were housed in a cage in a temperature-controlled (25 ℃) vivarium and food and water were provided ad libitum under a 12-h light/dark cycle with lights on at 07:00 am. Only male C57BL/6J mice were used in all experiment. All experimental procedures were approved by the Animal Care and Use Committee of Nanchang University (approval No.: ncdxsydwll-2018-26).

### Stereotaxic surgery

Four-week-old mice were anaesthetized with an intraperitoneal injection of 2% pentobarbital sodium and placed in a stereotaxic frame (RWD, Shenzhen, China). For labelling the BLA- and NAc-projecting mPFC neurons, the retrogradely AAV2/retro-hSyn-mCherry and AAV2/retro- hSyn-EGFP virus were bilaterally injected (0.2 μL per hemisphere) into the BLA (posterior to bregma, AP = − 1.28 mm; lateral to the midline, ML =  ± 3.2 mm; below the bregma, DV = − 5.05 mm) and NAc (AP =  + 1.42 mm; ML =  ± 0.85 mm; DV = − 4.7 mm) with a glass micropipette at a rate of 80 nl/min using a stereotactic injector (QSI, Stoelting, Wood Dale, IL, USA), respectively.

### Confocal imaging

Mice were deeply anesthetized with 2% pentobarbital sodium and transcardially perfused with PBS and of 4% paraformaldehyde (PFA). Brains were quickly removed from the skull and then post-fixed overnight at 4 ℃ in PFA. 40 μm thick coronal slices including the dmPFC or vmPFC were cut using VT1200S Vibratome (Leica Microsystems). Slices were washed in PBS 3 for 5 min and incubated with DAPI solution (Beyotime, China) for nuclear labeling and then mounted onto slides with Fluoromount aqueous mounting medium (Sigma-Aldrich, Sant Louis, Missouri, MO, USA). Images were taken using a confocal laser scanning microscope (Olympus FV1000, Tokyo, Japan). The number of virus-labeled neurons in dmPFC or vmPFC was calculated using ImageJ software (version 1.50, National Institutes of Health, Bethesda, USA) with the Cell Counter plug-in for assessing the overlay of BLA-projecting and NAc-projecting dmPFC or vmPFC neurons.

### Chronic restraint stress

Fifty-day-old mice were subjected to a restraint cylinder fitted closely to body size for 2 h per day at 10:00 am, for 10 consecutive days. For non-stressed control mice, they were transferred to the experimental room from their home cages and gently handled for 5 min before being returned to the holding room 2 h later.

### Chronic unpredictable stress

Fifty-day-old mice were subjected to a variety of stressors at different times of the day for 10 days. The stressors included 2-h restraint, 15-min tail pinch, 24-h constant light, 24-h wet bedding with 45° cage tilt, 10-min inescapable foot shocks, and social isolation. Stress exposure was conducted in a procedure room.

### Open field test

Each mouse was habituated to test room for 30 min prior to experimentation and placed in the center of a chamber, made of transparent plastic (50 × 50 cm), for 10 min with monitored by overhead video-tracking system (Med Associates Inc., Farifax, VT, USA). The maze was cleaned with 75% ethanol between each trial. ANY-maze software (Stoelting Co., USA) was used to analyze the time mice spent in center area and total distance they traveled during each test.

### Elevated plus maze test

Mice placed in the center of a plus-shaped maze with a pair of closed and open arms followed 30-min habituation in testing room. During behavioral test, a video-tracking system (Med Associates Inc., Farifax, VT, USA) was used to monitored their behavior for 10 min. The maze was cleaned with 75% ethanol between each trial. The ANY-maze software (Stoelting Co., USA) was used to analyze the time spent in open arms and entries into the open or closed arms.

### Electrophysiological slice recording

Mice were anesthetized with ether and decapitated, then their brains were rapidly removed and chilled in well-oxygenated (95% O_2_ and 5% CO_2_) ice-cold dissection buffer containing (in mM): 80 NaCl, 3.5 KCl, 4.5 MgSO_4_, 0.5 CaCl_2_, 1.25 NaH_2_PO_4_, 25 NaHCO_3_, 90 sucrose, and 10 glucose. Coronal brain slices (320 μm) containing dmPFC or vmPFC were cut using VT1200S microtome (Leica Microsystems) and were subsequently transferred to oxygenated ACSF containing (in mM): 124 NaCl, 2.5 KCl, 2 MgSO_4_, 2.5 CaCl_2_, 1.25 NaH_2_PO_4_, 22 NaHCO_3_, and 10 glucose, for 30 min recovery at 34 ℃. Then the brain slices were maintained at RT for at least 1 h and a single slice was transferred to the recording chamber and continuously perfused with oxygenated ACSF during all electrophysiological studies. Automatic temperature controller (TC-324B, Warner Instrument Co. Hamden, CT, USA) was used to maintain the temperature of ACSF in the chamber at 29 ± 1 ℃. Recording patch pipettes were made from filamented borosilicate glass capillary tubes (inner diameter, 0.84 μm) by using a horizontal pipette puller (P-97; Sutter Instrument Co., Novato, CA, USA). The recording of mEPSCs and mIPSCs was performed as previous study [37]. Briefly, tetrodotoxin (1 μM) was added in the bath solution and the patch pipettes were filled with an intracellular solution containing (in mM): 130 Cs-methanesulfonate, 5 NaCl, 1 MgCl_2_, 10 HEPES, 0.2 EGTA, 2 MgATP, 5 QX314, and 0.1 NaGTP, pH was adjusted to 7.30 with CsOH. To evoke action potentials, picrotoxin (100 μM) and CNQX (20 μM) were added in the bath solution. The virus-labelled neurons were injected with the depolarizing current pulses with their strength ranging from 0 to 250 pA and increased at a 50-pA step. The AP amplitude was measured as the voltage difference between the threshold and peak of the AP. The AP Half-width was determined as the duration of half-height between the threshold and the peak of the AP. The voltage sag ratio was calculated using the following equation: sag ratio = V_max_/V_ss_ = (V_baseline_ − V_min_)/(V_baseline_ − V_steady_), in which V_steady_ is the voltage averaged within 50 ms before the end of current injection, V_baseline_ is the resting membrane potential, and V_min_ is the hyperpolarizing current that induced minimum voltage. The AP rheobase was defined as the smallest current that produce the first spike. The patch pipettes were filled with 130 mmol/L k-gluconate. Data were collected with the PATCHMASTER software (version 2.53) using the patch-clamp amplifier (EPC 10 USB, HEKA Instrument, Ludwigshafen am Rhein, Germany).

### Statistical analyses

The statistical analyses were performed by GraphPad Prism (GraphPad Software, Inc., San Diego, CA, USA). Data were analyzed using Student t test or two-way analysis of variance with or without repeated measures, followed by post hoc test with Bonferroni correction. Data are presented as means ± SEM. The threshold for statistical significance was *p* < 0.05.

## Supplementary Information

**Additional** **file** **1:** **Figure** **S1**. Characterizations of the specificity of BLA- and NAc- projecting neurons in mPFC. **A** Representative images showing the co-labeling of mPFC→BLA PNsand pyramidal neuronal marker-CaMKIIα. Scale bar: 100 μm. **B** Summary plots showing the ratio of CaMKIIα expressing cells in mPFC→BLA PNs. **C** Representative images showing the colabeling of mPFC→NAc PNsand CaMKIIα. Scale bar: 100 μm. **D** Summary plots showing the ratio of CaMKIIα expressing cells in mPFC→NAc PNs. **Figure** **S2**. CUS significantly induces anxiety-like behavior in mice. **A** Experimental procedures. **B** Representative activity tracking in EPMT. **C, D** Summary plots of time in open arms  and open-arm entries  during EPMT. **E **Representative activity tracking in center area OFT. **F** Summary plots of time in center area during OFT. **G **Summary plots of total distance travelled during OFT. **H** Summary plots of mean speed during OFT. **Figure** **S3**. CUS markedly decreases inhibitory synaptic transmission onto dmPFC^→^^BLA^ PN in layer V. **A** Representative traces showing mEPSCs in dmPFC layer II/III. **B, C** Summary plots of averaged mEPSCs frequency  and amplitude . **D** Representative traces showing mEPSCs in dmPFC layer V. **E,F **Summary plots of averaged mEPSCs frequency  and amplitude . **G** Representative traces showing mIPSCs in dmPFC layer II/III. **H, I **Summary plots of averaged mIPSCs frequency  and amplitude. **J** Representative traces showing mIPSCs in dmPFC layer V. **K, L** Summary plots of averaged mIPSCs frequency and amplitude. **M** Summary plots of I/E frequency ratio in dmPFC layer II/III. **N** Summary plots of I/E amplitude ratio in dmPFC layer II/III. **O **Summary plots of I/E frequency ratio in dmPFC layer V. **P **Summary plots of I/E amplitude ratio dmPFC layer V. **Figure** **S4**. CUS does not changes synaptic transmission onto both vmPFC^→BLA^ or vmPFC^→NAc^ PN. **A** Representative traces showing mEPSCs in vmPFC layer II/III. **B, C **Summary plots of averaged mEPSCs frequency and amplitude. **D **Representative traces showing mEPSCs in vmPFC layer V. **E,F **Summary plots of averaged mEPSCs frequency and amplitude. **G** Representative traces showing mIPSCs in vmPFC layer II/III. **H, I **Summary plots of averaged mIPSCs frequency  and amplitude. **J** Representative traces showing mIPSCs in vmPFC layer V. **K, L **Summary plots of averaged mIPSCs frequency and amplitude. **M** Summary plots of I/E frequency ratio in vmPFC layer II/III. **N **Summary plots of I/E amplitude ratio in vmPFC layer II/III. **O **Summary plots of I/E frequency ratio in vmPFC layer V. **P **Summary plots of I/E amplitude ratio in vmPFC layer V.

**Additional** **file** **2:** Statistical detail informationfor figures.

## Data Availability

The data of this study are available in the paper and Additional files 1 and 2.
