# Supplementary material for: Circuit- and laminar-specific regulation of medial prefrontal neurons by chronic stress
Source: Cell Biosci. 2023 May 18;13:90. doi: 10.1186/s13578-023-01050-2 (PMC10197342; doi:10.1186/s13578-023-01050-2)
Supplement: Supplementary file 1 — Additional file 1: Figure S1. Characterizations of the specificity of BLA- and NAc- projecting neurons in mPFC. A Representative images showing the co-labeling of mPFC→BLA PNsand pyramidal neuronal marker-CaMKIIα. Scale bar: 100 μm. B Summary plots showing the ratio of CaMKIIα expressing cells in mPFC→BLA PNs. C Representative images showing the colabeling of mPFC→NAc PNsand CaMKIIα. Scale bar: 100 μm. D Summary plots showing the ratio of CaMKIIα expressing cells in mPFC→NAc PNs. Figure S2. CUS significantly induces anxiety-like behavior in mice. A Experimental procedures. B Representative activity tracking in EPMT. C, D Summary plots of time in open arms and open-arm entries during EPMT. E Representative activity tracking in center area OFT. F Summary plots of time in center area during OFT. G Summary plots of total distance travelled during OFT. H Summary plots of mean speed during OFT. Figure S3. CUS markedly decreases inhibitory synaptic transmission onto dmPFC→BLA PN in layer V. A Representative traces showing mEPSCs in dmPFC layer II/III. B, C Summary plots of averaged mEPSCs frequency and amplitude . D Representative traces showing mEPSCs in dmPFC layer V. E,F Summary plots of averaged mEPSCs frequency and amplitude . G Representative traces showing mIPSCs in dmPFC layer II/III. H, I Summary plots of averaged mIPSCs frequency and amplitude. J Representative traces showing mIPSCs in dmPFC layer V. K, L Summary plots of averaged mIPSCs frequency and amplitude. M Summary plots of I/E frequency ratio in dmPFC layer II/III. N Summary plots of I/E amplitude ratio in dmPFC layer II/III. O Summary plots of I/E frequency ratio in dmPFC layer V. P Summary plots of I/E amplitude ratio dmPFC layer V. Figure S4. CUS does not changes synaptic transmission onto both vmPFC→BLA or vmPFC→NAc PN. A Representative traces showing mEPSCs in vmPFC layer II/III. B, C Summary plots of averaged mEPSCs frequency and amplitude. D Representative traces showing mEPSCs in vmPFC layer V. E, [file 13578_2023_1050_MOESM1_ESM.docx]

**Additional file 1**

**
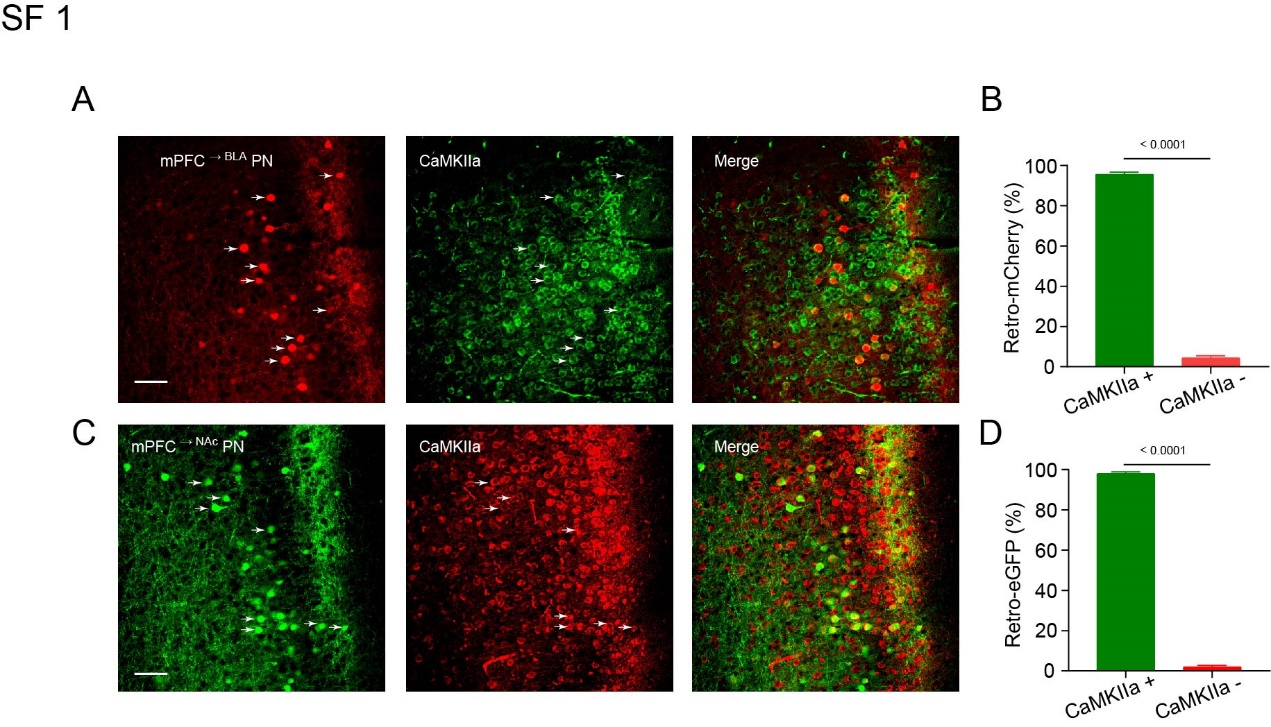
**

**SF 1. Characterizations of the specificity of BLA- and NAc- projecting neurons in mPFC. (A)** Representative images showing the co-labeling of mPFC^→BLA^ PNs (red) and pyramidal neuronal marker-CaMKIIα (green). Scale bar: 100 μm. **(B)** Summary plots showing the ratio of CaMKIIα expressing cells in mPFC^→BLA^ PNs. **(C)** Representative images showing the colabeling of mPFC^→NAc^ PNs (green) and CaMKIIα (red). Scale bar: 100 μm. **(D)** Summary plots showing the ratio of CaMKIIα expressing cells in mPFC^→NAc^ PNs.

**
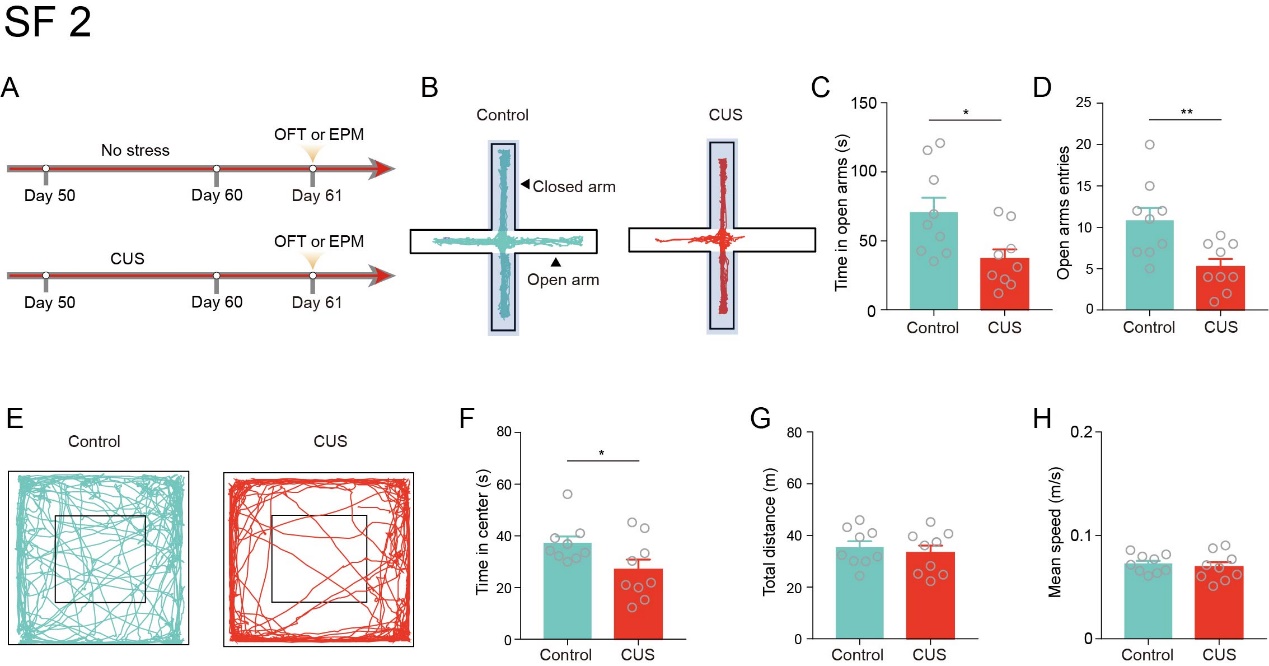
**

**SF 2. CUS significantly induces anxiety-like behavior in mice. (A)** Experimental procedures. **(B)** Representative activity tracking in EPMT. **(C, D)** Summary plots of time in open arms (C) and open-arm entries (D) during EPMT. **(E)** Representative activity tracking in center area OFT. **(F)** Summary plots of time in center area during OFT. **(G)** Summary plots of total distance travelled during OFT. **(H)** Summary plots of mean speed during OFT.


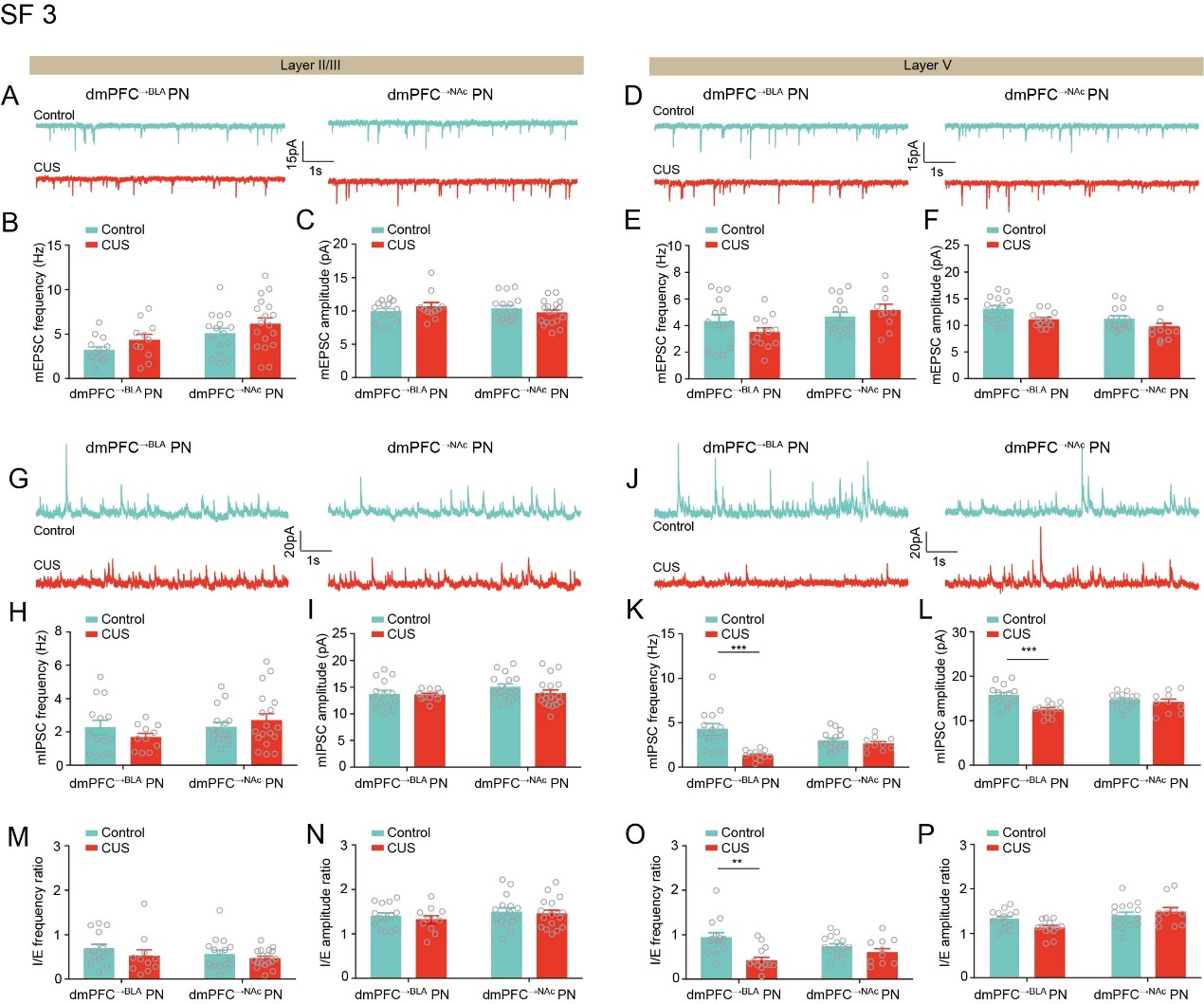


**SF 3. CUS markedly decreases inhibitory synaptic transmission onto dmPFC^→BLA^ PN in layer V. (A)** Representative traces showing mEPSCs in dmPFC layer II/III (scale bar: 1 s, 15 pA). **(B, C)** Summary plots of averaged mEPSCs frequency (B) and amplitude (C). **(D)** Representative traces showing mEPSCs in dmPFC layer V (scale bar: 1 s, 15 pA). **(E, F)** Summary plots of averaged mEPSCs frequency (E) and amplitude (F). **(G)** Representative traces showing mIPSCs in dmPFC layer II/III (scale bar: 1 s, 20 pA). **(H, I)** Summary plots of averaged mIPSCs frequency (H) and amplitude (I). **(J)** Representative traces showing mIPSCs in dmPFC layer V (scale bar: 1 s, 20 pA). **(K, L)** Summary plots of averaged mIPSCs frequency (K) and amplitude (L). **(M)** Summary plots of I/E frequency ratio in dmPFC layer II/III. (N) Summary plots of I/E amplitude ratio in dmPFC layer II/III. **(O)** Summary plots of I/E frequency ratio in dmPFC layer V. **(P)** Summary plots of I/E amplitude ratio dmPFC layer V.


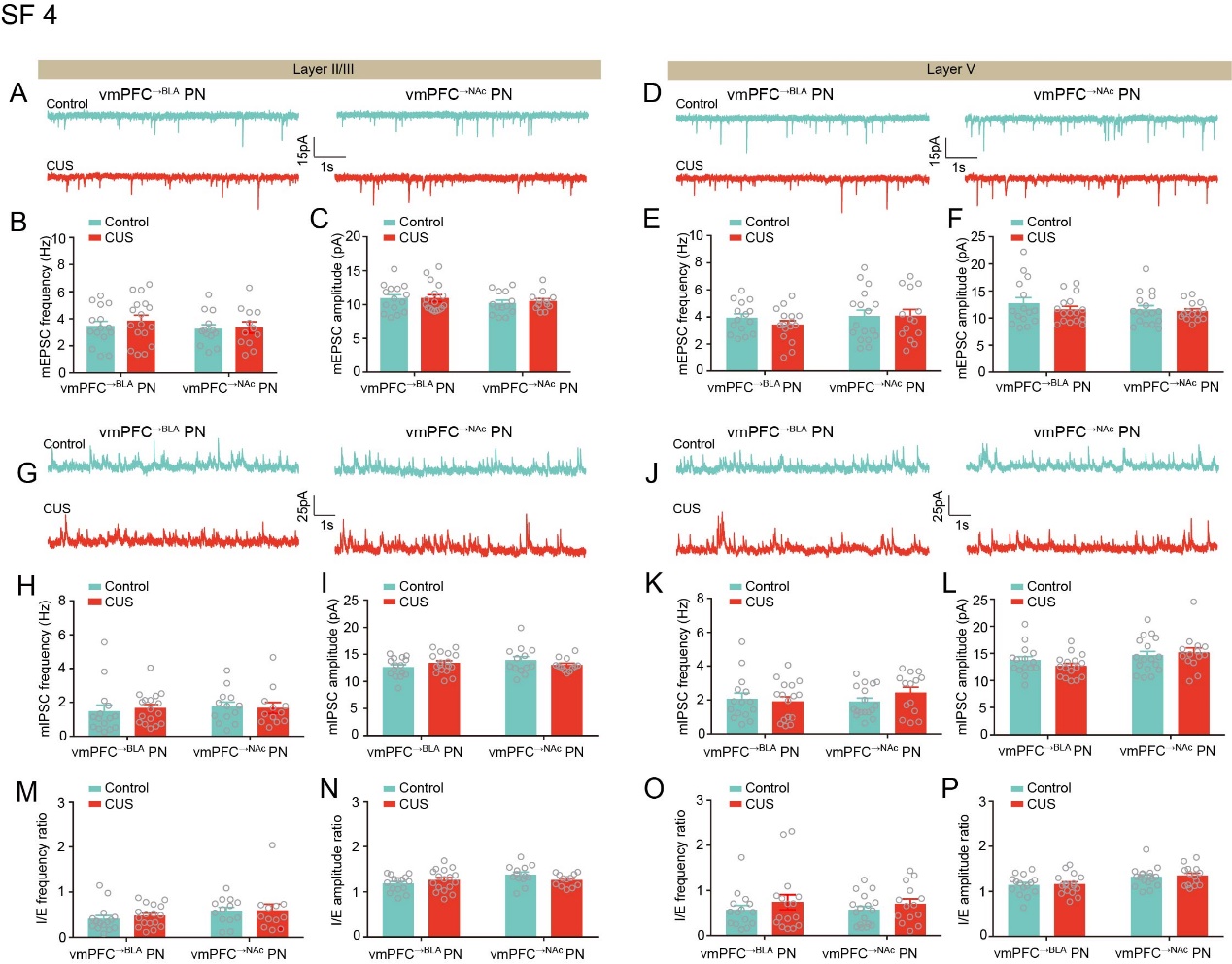


**SF 4. CUS does not changes synaptic transmission onto both vmPFC^→BLA^ or vmPFC^→NAc^ PN. (A)** Representative traces showing mEPSCs in vmPFC layer II/III (scale bar: 1 s, 15 pA). **(B, C)** Summary plots of averaged mEPSCs frequency (B) and amplitude (C). **(D)** Representative traces showing mEPSCs in vmPFC layer V (scale bar: 1 s, 15 pA). **(E, F)** Summary plots of averaged mEPSCs frequency (E) and amplitude (F). **(G)** Representative traces showing mIPSCs in vmPFC layer II/III (scale bar: 1 s, 25 pA). **(H, I)** Summary plots of averaged mIPSCs frequency (H) and amplitude (I). **(J)** Representative traces showing mIPSCs in vmPFC layer V (scale bar: 1 s, 25 pA). **(K, L)** Summary plots of averaged mIPSCs frequency (K) and amplitude (L). **(M)** Summary plots of I/E frequency ratio in vmPFC layer II/III. **(N)** Summary plots of I/E amplitude ratio in vmPFC layer II/III. **(O)** Summary plots of I/E frequency ratio in vmPFC layer V. **(P)** Summary plots of I/E amplitude ratio in vmPFC layer V.
